# Effects of In Vitro Digestion of Polyphenols from Coffee on Binding Parameters to Human Topoisomerase II α

**DOI:** 10.3390/molecules28165996

**Published:** 2023-08-10

**Authors:** Joanna Grzelczyk, Horacio Pérez-Sánchez, Miguel Carmena-Bargueño, Joanna Oracz, Grażyna Budryn

**Affiliations:** 1 Institute of Food Technology and Analysis, Faculty of Biotechnology and Food Sciences, Lodz University of Technology, 90-537 Lodz, Poland; grazyna.budryn@p.lodz.pl; 2 Structural Bioinformatics and High-Performance Computing Research Group (BIO-HPC), Computer Engineering Department, UCAM Universidad Católica de Murcia, Guadalupe, 30107 Murcia, Spain; hperez@ucam.edu (H.P.-S.); mcarmena@ucam.edu (M.C.-B.)

**Keywords:** coffee, TOPIIα, ITC, docking simulation, chlorogenic acids, in vitro digestion, cancer

## Abstract

Type II topoisomerase (TOPII) is an enzyme that influences the topology of DNA. DNA breaks generated by TOPII may result in mutagenic or cytotoxic changes in cancer cells. In this study, we characterized interactions of TOPIIα with coffee extracts and individual chlorogenic acids (CHAs) from the extracts by performing isothermal titration calorimetry (ITC) and molecular docking (MD) simulations. The study showed that the highest affinity to TOPIIα was found in green coffee (ΔG = −38.23 kJ/mol) and monochlorogenic acids fraction of coffee extracts (ΔG = −35.80 kJ/mol), resulting from the high content of polyphenols, such as CHAs, which can bind to the enzyme in the active site. Coffee extracts and their fractions maintained a high affinity for TOPIIα after simulated digestion in the presence of probiotic bacteria. It can be concluded that coffee may be a potential TOPIIα inhibitor considered as a functional food for cancer prevention.

## 1. Introduction

Topoisomerases are a special class of essential enzymes that manage the higher-order structural state of DNA. These enzymes are essential for the survival of all organisms by selectively cleaving, rearranging, and relegating DNA strands [1,2]. Topoisomerases help disentangle interlinked chromosomes and regulate the topological structure of the genetic material during cellular processes [2]. Topoisomerases can be separated into two general categories, termed type I or type II, based on the number of DNA strands of a single duplex cut during a catalytic cycle, respectively [3]. Type I topoisomerases transiently cleave a single strand of the DNA duplex, and can modulate DNA supercoiling, but cannot remove knots or tangles from intact duplex DNA. However, type II topoisomerases cleave both strands and can regulate DNA under- and over-winding, and can also remove tangles and knots from the genome [1,4,5]. Type II topoisomerases (TOPII) alter DNA topology, are involved in a number of critical nuclear processes in eukaryotic cells, such as DNA replication, recombination, and transcription, and are required for proper chromosome structure and segregation. However, the DNA breaks generated by TOPII can be subverted to give rise to mutagenic or cytotoxic lesions [5,6,7]. TOP II are enzymes that play essential roles in fundamental nuclear processes, and catalyze an ATP-dependent strand passing reaction. These enzymes have two isoforms: type IIα and IIβ [8]. The isozymes share 72% identity in their amino acid sequences, but differ greatly in their C termini and during cell growth and differentiation. TOPIIβ is expressed in all cell types irrespective of proliferation status and is involved in the transcription of hormonally regulated genes [8,9,10,11]. However, the biological function of IIβ is less characterized. TOPIIα is an essential enzyme that is expressed in proliferating cells and required for DNA replication, chromosome condensation, and decondensation, and can also play roles in transcription [12,13,14]. TOPIIα is a key nuclear enzyme for controlling the topological states of DNA by generating transient breakage in double-stranded DNA. It is encoded by the TOPIIα gene located on chromosome 17q12-q21 [15,16]. TOPIIα plays a critical role in chromosome instability and is overexpressed in tumor cells, which can activate the transcriptional functions of mutants [17,18,19,20,21].

The coffee bean has a high content of polyphenolic substances. Recently, interest in the biological effects of coffee polyphenols and caffeine has increased because of the potential health benefits. Additionally, in many epidemiological studies, coffee consumption has been associated with a reduced risk of several types of cancer [22,23,24]. In this study, bioactive compounds of coffee were studied for their ability to inhibit TOPIIα in in vitro models. We analyzed the interactions of TOPIIα with coffee extracts and individual chlorogenic acids (CHAs) from the extracts by performing isothermal titration calorimetry (ITC) and molecular docking (MD) simulation. In the next step, simulated in vitro digestion of coffee extracts was performed. The affinity for TOPIIα was again assessed after each digestion step. The aim of this study was to indicate the most stable enzyme-ligand complexes based on thermodynamic analyses and to determine the compounds in the studied coffee bean extracts that show the highest affinity for the enzyme depending on the roasting level and bioavailability.

## 2. Results and Discussion

### 2.1. Characterization of the Complexes of CHAs and Caffeine with TOPIIα Based on ITC

The thermodynamic parameters of interactions at constant temperature could be measured by ITC, which gives valuable information on protein–ligand complex formation [25]. The interactions of TOPIIα with coffee components in the first step of the study were analyzed using single bioactive substances. Integrated heat evolved per mole of coffee components titrated, corrected for the heat of their dilution, against the molar ratio of coffee components to TOPIIα gives an energy peak (kcal/s). During an ITC run, an exothermic (ΔH < 0) or endothermic (ΔH > 0) reaction is recorded, which occurs as a result of the binding of ligands at the active site or conformational rearrangement. According to the changes in enthalpy (ΔH) and entropy (ΔS) registered during the interactions, the prevailing specific type of the reaction can be determined, considering the values of ΔH and ΔS as follows: ΔH < 0 and ΔS < 0, van der Waals interactions; ΔH > 0 and ΔS > 0, hydrophobic forces and hydrogen bonds; and ΔH < 0 and ΔS > 0, electrostatic forces [25,26]. The changes in entropy in the system represent the degree of order/disorder of the system. A positive entropy change indicates an increase in the molecular disorder degree. 

The thermodynamic parameters of the interaction of CHAs with TOPIIα are shown in Table 1. The negative ΔH and positive ΔS values suggest that hydrogen bonds along with electrostatic forces played major roles in the binding of chlorogenic acids and their derivatives or caffeine to TOPIIα. The ΔG negative values indicate that the interactions were spontaneous. The affinity of bioactive compounds for TOPIIα decreased in the following order: ferulic acid > caffeic acid > 3,5-di-*O*-caffeoylquinic acid > caffeine > 3-*O*-caffeoylquinic acid > 5-*O*-caffeoylquinic acid > 4-*O*-caffeoylquinic acid > dihydrocaffeic acid > 4,5-di-*O*-caffeoylquinic acid. The ITC measurements did not show significant interactions with 5-(hydroxymethyl)furfural and acrylamide, the compounds formed in coffee beans during the roasting process. Among the studied compounds, ferulic acid exhibited the highest affinity for TOPIIα and the enthalpy change during interactions (ΔG: −71.18 kJ/mol and ΔH: −17.33 kJ/mol, respectively) (Table 1). The 4,5-di-*O*-caffeoylquinic acid was also characterized by the high binding affinity for TOPIIα (−37.64 kJ/mol), but the enthalpy change (ΔH = −1.78 kJ/mol) was significantly lower than other CHAs or caffeine. The higher enthalpy change of interactions of other CHAs with the enzyme during titration may be due to the more efficient formation of the pi-pi interactions of the aromatic ring of the caffeoyl moiety with TOPIIα residues, which in the case of 4,5-di-*O*-caffeoylquinic acid may be distorted by spatial mismatch. 

**Figure 1:**
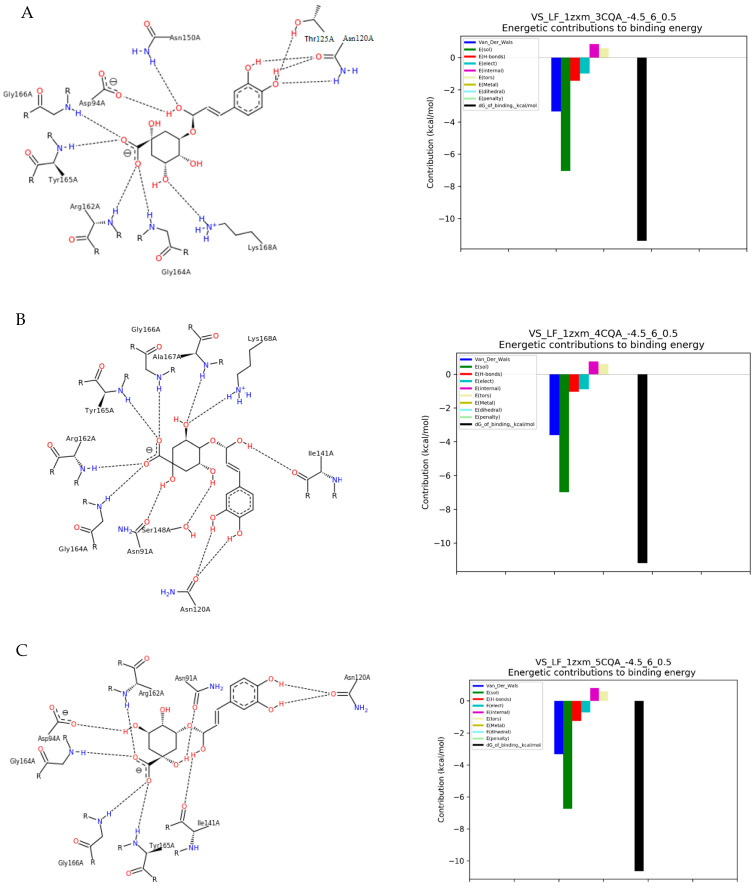

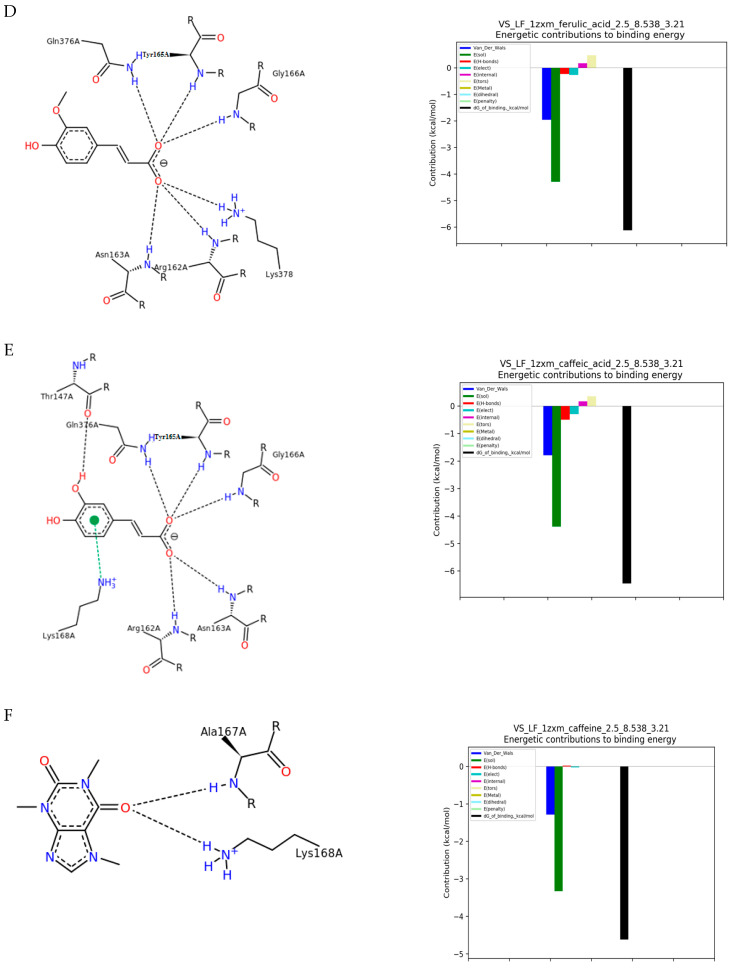

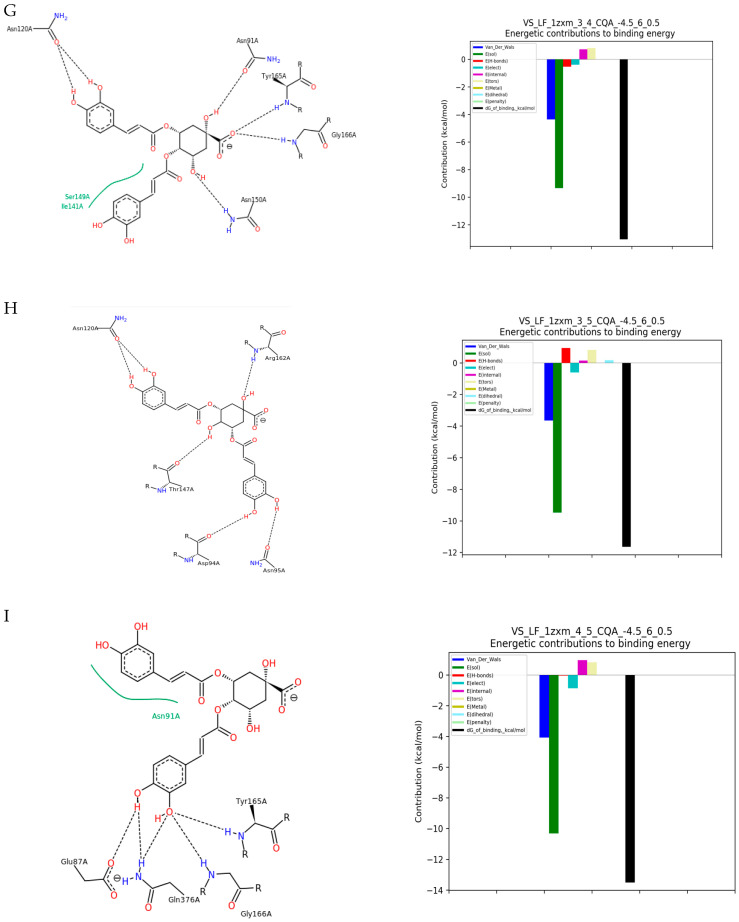
The results of docking simulation. On the left, a 2D model of the ligand interaction with TOPIIα. On the right, the energy values of the interactions that make up the total binding energy of the ligand with TOPIIα. (**A**) 3-CQA (3-*O*-caffeoylquinic acid); (**B**) 4-CQA (4-*O*-caffeoylquinic acid); (**C**) 5-CQA (5-*O*-caffeoylquinic acid); (**D**) Ferulic acid; (**E**) Caffeic acid; (**F**) Caffeine; (**G**) 3,4-DiCQA (3,4-di-*O*-caffeoylquinic acid); (**H**) 3,5-DiCQA (3,5-di-*O*-caffeoylquinic acid); (**I**) 4,5-DiCQA (4,5-di-*O*-caffeoylquinic acid).

It is worth noting that ferulic acid bound as a competitive inhibitor (Figure 1D). This was observed in the present study as well as the study by Lu et al. [27], who reported that ferulic acid can act as a competitive inhibitor of TOP at a concentration of 10–30 µM. The cited study showed that ferulic acid can inhibit cell proliferation in *Procaryote* by directly binding the DNA-gyrase complex [27]. Additionally, Bandele et al. [28] reported that polyphenols are a diverse and complex group of compounds that interact with the enzyme in a noncovalent manner but can also react by covalent adduction. They tested two classes of polyphenols (catechins and flavanols) against human topoisomerase IIα and showed that 100 μM solutions of quercetin, kaempferol, or myricetin acted as redox-dependent poisons rather than the enzyme inhibitors. According to Quideau et al. [29], grape-derived polyphenols contained in wine and ellagitannins derived from oak barrels may form condensation products that inhibit the activity of human TOPIIα. Thus, the inhibition of TPOIIα activity of phenolic acids has been much less frequently investigated in this type of study compared to flavonoids.

### 2.2. Characterization of the Complexes of CHAs and Caffeine with TOPIIα Based on MD

TOPIIα inhibitors are categorized into two classes. The first type can stabilize the TOPII α-DNA cleavage complex, leading to DNA double-strand breaks [30,31]. The other type, unlike TOPIIα, acts by inhibiting the catalytic activity of TOPIIα without DNA break generation [5,6,31]. The structures of TOPIIα can be divided into three domains based on sequence homology with the bacterial type II enzyme, DNA gyrase [31,32]. The N-terminal domain is homologous to the B-subunit of DNA gyrase and contains the site of ATP binding and hydrolysis [30,32,33]. The central domain is homologous to the A-subunit of DNA gyrase and contains the active site tyrosine required for DNA cleavage and ligation [5,34,35]. The A domain was considered for coffee bioactive compounds binding in our study. The C-terminal domain contains nuclear localization sequences and sites of phosphorylation [6,36].

To evaluate the binding modes and affinities between coffee bioactive components and TOPIIα, we performed molecular docking simulations using all the moieties as ligands. Their binding modes are shown in Figure 1. 

We can observe that the strongest affinity for the enzyme calculated from the molecular docking was observed for dichlorogenic acids. Molecular docking with TOPIIα revealed the highest binding affinity of −57.35 kJ/mol for 4,5-di-*O*-caffeoylquinic acid (Table 1, Figure 1I), which exhibited strong hydrogen bindings with TOPIIα. It formed five H-bonds with the five catalytic residues including Tyr165A, Glu87A, Gln376A (two different configurations), and Gly166A of the TOPIIα. Additionally, MD indicated one hydrophobic interaction with Asn91A. A slightly lower binding affinity was shown for 3,4-di-*O*-caffeoylquinic acid (−51.33 kJ/mol, Figure 1G) and 3,5-di-*O*-Caffeoylquinic acid (−49.40 kJ/mol). 3,4-di-*O*-caffeoylquinic acid formed four H-bonds with six amino acids acting as catalytic residues: Tyr165A, Asn91A, Asn150A, and Asn120A (two different configurations in the ring), and Gly166, as well as two hydrophobic interactions with Ser149 and Ile141A. Whereas, 3,5-di-*O*-caffeoylquinic acid interactions with the enzyme resulted in six H-bonds with four residues: Thr147A, Asn95A, Asn120A (two different configurations in the ring), and Asp126A (Figure 1H).

The monochlorogenic acids (isomers of caffeoylquinic acid) showed a difference in affinity for TOPIIα compared to diesters (isomers of dicaffeoylquinic acid), in the range of −42.70 to −44.38 kJ/mol (Table 1). This relationship was related to the energy of both salt bridges and hydrogen bonds formed during interactions, but not directly to their number (Figure 1). The highest affinity for the enzyme was found for 4-CQA, derived from the formation of ten hydrogen bonds with Arg162A, Asn120A, Asn91A, Tyr165A, Lys168A, Gly164A, Gly166A, Ala167A, Ser148A, and Ile141A (Figure 1B). The isomerization of the ester bond in 3-CQA resulted in different H-bonds formation with eight residues: Arg162A, Asn150A, Asn120A, Tyr165A, Lys168A, Gly164A, Gly166A, and Asp94A (Figure 1A). 5-CQA formed nine H-bonds with the amino acids of catalytic residues: Tyr165A, Asn120A, Asn150A, Arg162A, Asp94A, Thr215A, Lys168A, Gly164A, and Gly166A. 

Molecular docking of ferulic acid to TOPIIα showed the affinity amounting to −25.96 kJ/mol (Table 1). The energetic changes showed interactions with Gln376A, Gly166A, Lys378A, Asn163A, Arg162A, and Tyr165A of the catalytic pocket (Figure 1D). The caffeic acid interactions with TOPIIα displayed the affinity score of −26.37 kJ/mol (Table 1) and mainly showed hydrogen bonds formation with Thr147A, Arg162A, Asn163A, Tyr165A, Lys168A, Gln376A, Gly164A, and Gly166A (Figure 1E). In addition to these interactions, pi-pi attractions with the amino acid residue Lys168A also occurred. Molecular docking of caffeine with TOPIIα revealed the lowest binding affinity of −19.28 kJ/mol among the studied compounds (Table 1). The alkaloid was found to form two hydrogen bonds with Ala167A and Lys168A of moderate energy value (Figure 1F).

The binding energy values obtained using ITC and molecular docking in the case of the following acids confirm the spontaneous type of the reaction and binding in the active site of TOPIIα: 3-CQA, 4-CQA, 5-CQA, 3,4-diCQA, 3,5-diCQA, and 4,5-diCQA. The differences in the energy parameters determined by the two methods result from the greater specificity of MD, where ligands are docked only to the active site, as opposed to ITC, where the interactions take place on the entire available surface of the protein, and additionally, ITC test parameters may reflect conformational changes of molecules. In conclusion, both methods provide important information about the interactions. Within both methods, the compound with the highest potential for enzyme activity inhibition appears to be 4,5-diCQA.

### 2.3. Characterization of the Interactions of Coffee Extracts before and after In Vitro Digestion with TOPIIα Based on ITC

The analysis of individual substances characteristic of coffee beans showed a high affinity for the enzyme. In the next step of the work, the coffee extracts were interacted with the enzyme, before and after in vitro digestion in a simulated digestive system, to verify the hypothesis that a complex mixture of bioactive compounds could give a higher enzyme activity inhibition effect related to binding more than one ligand from the extract and thus blocking the catalytic site more efficiently. The values of the interaction parameters such as ΔG, ΔH, and ΔS obtained after fitting are listed in Table 2.

Coffee extracts before and after roasting showed the exothermic type of interactions with TOPIIα. They showed high affinity for the enzyme before digestion. Stronger complexes were formed with Robusta, while Arabica showed weaker binding to TOPIIα. Interestingly, in Arabica coffee, the degree of roasting did not statistically differentiate the binding effects. The highest affinity was shown by green Robusta coffee extract with a value of −38.23 kJ/mol (ΔG), and the lowest was recorded for all Arabica extract, about −33 kJ/mol (ΔG). The same dependence was observed for the enthalpy of the interactions.

After the simulated digestion of coffee extracts in the stomach, we can observe a decrease in the reaction enthalpy and the affinity for the enzyme in the case of Arabica, while Robusta, especially green, increased enthalpy changes. With the digestion progress, the enthalpy of the reaction decreased, while the affinity increases significantly (Table 2). Interestingly, the addition of probiotic bacteria during digestion in the large intestine had a positive effect on increasing both the affinity and enthalpy of interactions. Regardless of the stage of digestion, Robusta showed more favorable parameters of binding to TOPIIα, which is related to the higher content of chlorogenic acids of this species. In our previous study, we analyzed the bioavailability of coffee compounds during digestion measured as a concentration of the individual polyphenols (UHPLC-MS chromatography was used for the qualitative and quantitative determination of compounds) and showed that the content of ferulic and caffeic acids increases as a result of hydrolysis of ester bonds characteristic for chlorogenic acids [37]. Alwes et al. in their work [38] reached similar conclusions that caffeic and ferulic acids become the dominant phenolics during coffee in vitro digestion. From Table 1, we can observe that ferulic acid and caffeic acid have a high affinity for the enzyme. 

### 2.4. Characterization of the Interactions of Fractions Obtained from Coffee Extracts before after In Vitro Digestion with TOPIIα Based on ITC and MD

Coffee extracts could be fractionated and used as bioactive preparations for example for functional food supplementation with antioxidants. In order to compare coffee extracts with the bioactive preparations obtained by fractionation and partial purification of coffee extracts as TOPIIα inhibitors, the obtained fractions, i.e., monochlorogenic and dichlorogenic acids and caffeine, were subjected to in vitro digestion and ITC analysis. Fractions were isolated from coffee extracts using the CPC technique and contained some amounts of other compounds. Fractionation and digestion of preparations from the coffee extracts were described in our previous work [39]. Coffee fractions were subjected to ITC analysis (Table 3) and also to molecular docking (mixture of the main compounds of the fraction) (Table 4).

Fractions obtained from coffee extracts were partially purified and contained free CHAs as well as CHAs bound to proteins and hydrocarbons. In the case of the fractions obtained from roasted beans, the affinity increased significantly with light and dark roasting for caffeine preparation and then remained at a high level. However, the highest affinity for TOPIIα was determined for monochlorogenic acids from Robusta green coffee (ΔG = −35.80 kJ/mol), accompanied by the highest reaction enthalpy (ΔH = −2.40 kJ/mol (Table 3). After the first stage of digestion, the enthalpy of the complexation decreased for the fraction of monochlorogenic acids isolated from Robusta and Arabica, from the range of −2.40–−0.75 kJ/mol to −1.63–−0.48 kJ/mol, and for the fraction of dichlorogenic acids from the range of −1.44–−0.52 kJ/mol to −1.83–−0.34 kJ/mol. In the case of caffeine, from about −1 kJ/mol it decreased during digestion for dark roasted coffees, while for green and light roasted coffees the enthalpy of the reaction increased slightly. Using additionally the enzymatic activity of probiotic bacteria in the last stages of digestion, a renewed increase in the enthalpy changes accompanying the interactions was observed, which was not the case in parallel in vitro digestion tests without bacteria. We can observe a similar relationship for the affinity of preparations for the enzyme recorded during ITC experiments. The highest values occurred for fractions digested with bacteria. Considering the molecular docking, we can see that ∆G was twice as large as for single substances. This shows that the mixture of bioactive compounds contained in fractions can bind to the enzyme in the active site using a higher number of amino acids residues, which was confirmed by the molecular docking of mixtures of CHAs with the enzyme (Table 4). The MD showed a high affinity for the enzyme for dichlorogenic acids of −48.70 to −56.53 kJ/mol (Table 4). This confirms the high affinity of diCQAs for the enzyme recorded in the tests of individual CHAs. The sequence of docking in the use of mixtures was considered, which revealed statistically important differences of ∆G. The practical significance of this observation concerns the fact that the concentration of individual CHAs in the preparations, which may depend on the type of coffee and the method of extract purification, may affect the inhibition of enzyme activity. This stage of the study confirmed the high activity of 4,5-diCQA, which in the mixture was comparable in the activity with this compound analyzed individually, assuming that in the mixture it docks first, which is most likely when it is present in the highest concentration.

The bond length between the ligand and the protein was in the range of 1.83–3.34 Å in the case of H-bonds, and statistically longer for salt bridges, which were identified in the number of one or two bonds, depending on the mixture considered. 

## 3. Materials and Methods

### 3.1. Chemicals and Materials

Topoisomerase II α human, 3-caffeoylqunic acid (≥99%), 4-caffeoylqunic acid (≥99%), 5-caffeoylquinic acid (≥99%), caffeic acid (≥99%), caffeine (≥99%), acrylamide (≥99%), ferulic acid (≥99%), dihydrocaffeic acid (≥99%), 5-HMF (≥99%), 3,5-dicaffeoylquinic acid (≥99%) and 4,5-dicaffeoylquinic acid (≥99%), pepsin, pancreatin from porcine pancreas, porcine bile extract, mucin, tris-(hydroxy-methyl) aminomethane, 3,5-dinitrosalicylic acid, trinitrobenzenesulfonic acid, glucose, L-leucine, and α-amylase were purchased from Sigma Aldrich (St. Louis, MO, USA), and nylon filters were sourced from Chromacol (Herts, UK).

Research was carried out with two species of coffee beans: green Arabica (Brazil Cerrado), and green Robusta (India Cherry) purchased from Bero Polska (Gdynia, Poland). The methods of coffee roasting, extraction, fractionation, and digestion were described in the previous works [37,40]. In short, green, light roasted (195 °C, 6 min), and dark roasted coffee (230 °C, 6 min) was ground and then aqueous extracts were prepared (110 °C, 1.2 MPa). Subsequently, the extracts were freeze-dried and digested in a simulated digestive system without and in the presence of probiotics. The extracts, from which lyophilisates were obtained, were fractionated using the CPC method (Spot Prep II system (Armen, France)). The obtained coffee fractions, after ethanol and ethyl acetate evaporation, were subjected to freeze-drying, followed by digestion [41]. Samples taken after the given stages of in vitro digestion were frozen at −80 °C for analysis.

In short, the content of polyphenols: Robusta coffee extracts showed the content of hydroxycinnamic acids, including chlorogenic acids (CHAs) in the range of 5.71–31.45 g/100 g s.s., and Arabica 3.51–17.60 g/100 g s.s. With the increase in the degree of roasting in both types of coffee, there was a decrease in the content of CHAs, as well as, to a lesser extent, caffeine in the extracts. Robusta coffee contained 6.06 ÷ 6.10 g of caffeine/100 g s.s., and Arabica 3.90–4.41 g of caffeine/100 g s.s. However, the content of 5-HMF and AK increased during roasting [37].

Three fractions were collected from the green and roasted coffees: the caffeine fraction, where the alkaloid constituted from 10.01 to 21.90 g/100 g d.b.; the monochlorogenic acids, which contained caffeoyl- and feruloylquinic acids isolated from green and roasted coffees in the range of 8.91–50.41 g/100 g d.b. of the fraction; and the dichlorogenic acids, containing dicaffeoylquinic acids in the range of 3.09–22.33 g/100 g d.b [40].

An increase in the content of free polyphenols in extracts in the final stage of in vitro digestion was shown by 2 and 7% (respectively, in the case of green Robusta and Arabica extracts), while in the case of light roasted coffee the increase was as much as 161 and 256%, respectively, and in the case of dark roasted coffee 490 and 725%, compared to the content of polyphenols in the extracts before digestion [37]. Coffee fractions showed a similar tendency to release KHC as coffee extracts. The increase in the content of monochlorogenic acids in the last stage of digestion of green coffee was 134–136%, light roasted coffee was approx. 164%, and dark roasted coffee was 192–198% compared to the material before digestion. The content of dichlorgenic acids increased by 164–290% (green), by approx. 188% (light roasted) and 220–304% (dark roasted), respectively. The concentration of caffeine in the isolated fraction rich in this compound from coffee extracts was slightly reduced as a result of digestion; in the case of green and roasted coffee extracts, by 8 and 5%, respectively [39].

During the in vitro digestion of green Arabica coffee, the content of accompanying bacteria decreased by about 45% on average, while in the case of Robusta, it decreased by 50–55%. With the increase in the degree of coffee roasting, a decrease in the survival rate of lactic acid bacteria was demonstrated [37]. Fractions isolated from coffee extracts caused the highest survival in the case of dichlorogenic acids, reducing the number by 42%, while the remaining fractions reduced the number by more than 50% [39].

### 3.2. Isothermal Titration Calorimetry (ITC)

The parameters of interactions of coffee and its components with TOPIIα were carried out using the isothermal titration calorimetry (ITC) (MicroCal PEAQ-ITC 200, Malvern, Worcestershire, UK). Analyses were carried out according to Grzelczyk et al. [40], with some modifications. A calorimeter cell with a capacity of 0.2 mL was filled with 0.1 μmol/L of TOPIIα solution in ultrapure water (deionized, LC-MS grade); the syringe was subsequently filled with titrants as degassed aqueous solutions of chlorogenic acids (CHAs); green, light-, or dark-roasted coffee extracts; coffee fraction; before and after in vitro digestion, at concentrations of 10 mmol/L (coffee extracts at concentrations of 0.1 mmol/L calculated based on 5-caffeoylquinic acid). The measurements were carried out at 36.6 °C with continuous stirring at 307 rpm (10 injections, 25 min). During the ITC analysis, an exothermic or endothermic reaction was recorded, when the binding of compounds to the enzyme occurred. The heat was generated as a consequence of ligand-to-protein binding, which is observed as an energy peak (raw data of heat in kcal/s). The titration of a ligand into water was performed to subtract the energetic effects of dilution. The enthalpy changes (∆H), Gibbs free energy changes (∆G), and entropy changes (∆S) were calculated from the ITC titration nonlinear least squares saturation curve fitting carried out using MicroCal PEAQ-ITC200 software 1.30 [40].

### 3.3. Docking Simulation

Molecular docking (MD) simulations of the interactions between TOPIIα and bioactive compounds from coffee beans allow for a prediction of the interactions at the atomic level (hydrogen bonds, hydrophobic and van der Waals interactions, salt bridges, and the affinity of reagents). This enables the indication of the most preferable and stable complexes of bioactive compounds with TOPIIα enzyme involved in the cell cycle progression [42]. Three dimensional X-ray crystal structure of human DNA topoisomerase II, alpha isoenzyme (pdb id: 1zxm, resolution  =  1.87 Å, Chains: A, B) was downloaded from the protein data bank (PDB; http://www.rcsb.org/pdb, accessed on 10 July 2023). The modeling involved three steps. In the first stage, a full-atom model of the enzyme was prepared by calculating the ionizable amino acids’ pKa values and protonation states. Then, hydrogen atoms were added, and the cap termini were included with the Protein Preparation Wizard module available in Maestro. The charges of the atoms of the protein were added using AutoDockTools. The chemical structures of all bioactive coffee compounds were built up manually, and partial charges were calculated as previously reported to be used during docking simulations [43]. The docking of bioactive coffee compounds to the prepared enzyme structure model was performed with the AutoDock Vina docking software 1.1.2, using the default configuration parameters. We could indicate the hydrogen bonds (H-bonds), hydrophobic stabilization, electrostatic interactions, salt bridges, entropic penalty due to the number of rotatable bonds, and the internal energy of the ligand.

### 3.4. Statistical Analysis

The results were expressed as mean ± standard error of the mean, *n* = 6. All calculations were evaluated for significance using one-way ANOVA followed by Dunnett’s test with the GraphPad Prism 6.0 software (GraphPad Software, Inc., La Jolla, CA, USA). Significance was defined at *p* ≤ 0.05.

## 4. Conclusions

The ITC analysis showed that coffee extracts and preparations obtained after fractionation and partial purification are a source of natural, non-toxic compounds potentially capable of inhibiting topoisomerase IIα activity in vivo. Robusta green coffee extract showed the highest affinity for the enzyme resulting from the high content of polyphenols, such as CHAs, which bind to the enzyme at the active site confirmed by molecular docking. Caffeine showed the lowest affinity for TOPIIα, which suggests that chlorogenic acids mainly influence binding to the enzyme. Conducted tests using isothermal titration calorimetry to analyze the affinity of coffee extracts to TOPIIα after simulated in vitro digestion showed that the activity of the digested coffee extracts as well as their fractions, especially in the presence of probiotic bacteria, could be considered effective inhibitors of this enzyme in vivo. This preliminary study has shown that coffee is characterized by a high content of bioactive compounds with a high affinity for TOPIIα, which makes it possible to be a potential nutraceutic that can be used as a functional food supporting anticancer therapies.

## Figures and Tables

**Table 1:** Thermodynamic parameters of interactions between TOPIIα and hydroxycinnamic acids, caffeine, 5-(hydroxymethyl)furfural, or acrylamide.

Compound	∆H (kJ/mol) ITC	∆G (kJ/mol) ITC	∆G (kJ/mol) Docking Simulation	∆S (J/mol×K) ITC
Caffeic acid	−9.04 ± 0.25 ^a^	−33.54 ± 1.09 ^a^	−26.37	0.08 ± 0.0 ^a^
Ferulic acid	−17.33 ± 0.15 ^b^	−71.18 ± 1.15 ^b^	−25.96	0.02 ± 0.00 ^b^
Monochlorogenic acids				
3-*O*-Caffeoylquinic acid	−9.09 ± 0.35 ^a^	−33.54 ± 1.25 ^a^	−43.96	0.08 ± 0.02 ^a^
4-*O*-Caffeoylquinic acid	−9.17 ± 0.15 ^a^	−33.62 ± 1.33 ^a^	−44.38	0.08 ± 0.01 ^a^
5-*O*-Caffeoylquinic acid	−9.21 ± 0.35 ^a^	−33.49 ± 1.39 ^a^	−42.70	0.08 ± 0.01 ^a^
Dichlorogenic acids				
3,5-Di-*O*-caffeoylquinic acid	−8.00 ± 0.45 ^c,a^	−33.87 ± 1.39 ^a^	−49.40	0.08 ± 0.02 ^a^
4,5-Di-*O*-caffeoylquinic acid	−1.78 ± 0.15 ^d^	−37.64 ± 1.35 ^c^	−57.35	0.12 ± 0.02 ^c^
Dihydrocaffeic acid	−6.49 ± 0.29 ^e^	−34.42 ± 1.33 ^d^	−51.33	0.09 ± 0.03 ^d^
	Other compounds			
Caffeine	−6.28 ± 0.45 ^e^	−34.50 ± 1.15 ^d^	−19.28	0.09 ± 0.01 ^d^
5-(Hydroxymethyl)furfural	nd	nd	-	nd
Acrylamide	nd	nd	-	nd

Values are expressed as mean value ± SD; *n* = 6; ^a–e^ different letters in one column or no index correspond to significant differences (*p* < 0.05); nd: not detected.

**Table 2:** Thermodynamic parameters of interactions between TPOIIα and coffee extracts before and after in vitro digestion measured by ITC.

Type of Coffee Extract	∆H (kJ/mol)	∆G (kJ/mol)	∆S (J/mol×K)
Before in vitro digestion			
Green Arabica	−9.59 ± 0.49 ^a^	−33.41 ± 1.45 ^a^	0.32 ± 0.09 ^a^
Light roasted Arabica	−9.21 ± 0.39 ^a^	−33.49 ± 1.39 ^a^	0.33 ± 0.02 ^a^
Dark roasted Arabica	−9.63 ± 0.33 ^a^	−33.45 ± 1.35 ^a^	0.32 ± 0.06 ^a^
Green Robusta	−13.82 ± 0.39 ^b^	−38.23 ± 1.33 ^b^	0.33 ± 0.01 ^a^
Light roasted Robusta	−10.06 ± 0.35 ^c^	−33.62 ± 1.39 ^a^	0.32 ± 0.02 ^a^
Dark roasted Robusta	−10.37 ± 0.35 ^c^	−34.92 ± 1.39 ^a^	0.33 ± 0.03 ^a^
Gastric Phase			
Green Arabica	−7.45 ± 0.33 ^d^	−31.82 ± 1.49 ^c^	0.08 ± 0.00 ^b^
Light roasted Arabica	−7.29 ± 0.39 ^d^	−31.40 ± 1.35 ^c^	0.08 ± 0.00 ^b^
Dark roasted Arabica	−7.24 ± 0.25 ^d^	−31.61 ± 1.22 ^c^	0.08 ± 0.01 ^b^
Green Robusta	−26.17 ± 1.05 ^e^	−34.16 ± 1.15 ^a^	0.03 ± 0.00 ^c^
Light roasted Robusta	−13.94 ± 0.55 ^b^	−34.12 ± 1.15 ^a^	0.07 ± 0.01 ^c^
Dark roasted Robusta	−7.33 ± 0.35 ^d^	−34.04 ± 1.45 ^a^	0.09 ± 0.01 ^b^
Small Intestine Phase			
Green Arabica	−1.63 ± 0.49 ^e^	−37.56 ± 1.10 ^b^	0.12 ± 0.01 ^d^
Light roasted Arabica	−1.62 ± 0.35 ^e^	−37.22 ± 1.09 ^b^	0.11 ± 0.00 ^d^
Dark roasted Arabica	−1.58 ± 0.05 ^e^	−31.61 ± 1.09 ^c^	0.10 ± 0.02 ^d^
Green Robusta	−2.37 ± 0.15 ^f^	−38.48 ± 1.33 ^b^	0.12 ± 0.00 ^d^
Light roasted Robusta	−1.79 ± 0.15 ^e^	−38.31 ± 1.33 ^b^	0.12 ± 0.01 ^d^
Dark roasted Robusta	−1.71 ± 0.25 ^e^	−38.18 ± 1.25 ^b^	0.12 ± 0.01 ^d^
Large Intestine Phase after 4 h			
Green Arabica	−1.09 ± 0.49 ^e^	−37.56 ± 1.19 ^b^	0.12 ± 0.01 ^d^
Light roasted Arabica	−0.96 ± 0.29 ^g^	−35.71 ± 1.2 ^a^	0.11 ± 0.01 ^d^
Dark roasted Arabica	−0.81 ± 0.15 ^g^	−33.83 ± 1.34 ^c^	0.11 ± 0.00 ^d^
Green Robusta	−2.11 ± 0.49 ^f^	−40.07 ± 1.05 ^d^	0.12 ± 0.01 ^d^
Light roasted Robusta	−1.88 ± 0.35 ^e^	−39.61 ± 1.15 ^d^	0.12 ± 0.01 ^d^
Dark roasted Robusta	−1.79 ± 0.39 ^e^	−37.85 ± 1.22 ^a^	0.12 ± 0.01 ^d^
Large Intestine Phase after 10 h			
Green Arabica	−0.92 ± 0.10 ^g^	−36.51 ± 2.35 ^b^	0.11 ± 0.00 ^d^
Light roasted Arabica	−0.88 ± 0.05 ^g^	−36.34 ± 2.33 ^b^	0.11 ± 0.00 ^d^
Dark roasted Arabica	−0.79 ± 0.03 ^g^	−33.49 ± 2.25 ^a^	0.11 ± 0.00 ^d^
Green Robusta	−1.77 ± 0.15 ^e^	−39.90 ± 2.05 ^a^	0.12 ± 0.01 ^d^
Light roasted Robusta	−1.70 ± 0.33 ^e^	−38.23 ± 2.15 ^b^	0.12 ± 0.03 ^d^
Dark roasted Robusta	−1.01 ± 0.19 ^e^	−38.10 ± 2.09 ^b^	0.12 ± 0.02 ^d^
Large Intestine Phase + probiotic bacteria after 4 h			
Green Arabica	−1.68 ± 0.19 ^e^	−36.93 ± 1.49 ^b^	0.11 ± 0.01 ^d^
Light roasted Arabica	−1.64 ± 0.33 ^e^	−36.38 ± 1.33 ^b^	0.11 ± 0.01 ^d^
Dark roasted Arabica	−1.58 ± 0.59 ^e^	−35.80 ± 1.95 ^b^	0.11 ± 0.02 ^d^
Green Robusta	−9.13 ± 1.05 ^a^	−42.33 ± 2.25 ^d^	0.11 ± 0.00 ^d^
Light roasted Robusta	−6.36 ± 0.45	−42.20 ± 2.19 ^d^	0.12 ± 0.01 ^d^
Dark roasted Robusta	−2.14 ± 0.15 ^f^	−41.87 ± 2.05 ^d^	0.13 ± 0.00 ^d^
Large Intestine Phase + probiotic bacteria after 10 h			
Green Arabica	−1.67 ± 0.45 ^e^	−35.34 ± 1.15 ^b^	0.11 ± 0.00 ^d^
Light roasted Arabica	−1.61 ± 0.49 ^e^	−34.00 ± 1.49 ^c^	0.10 ± 0.00 ^d^
Dark roasted Arabica	−1.60 ± 0.35 ^e^	−33.87 ± 1.35 ^c^	0.10 ± 0.02 ^d^
Green Robusta	−8.16 ± 0.55 ^d^	−41.66 ± 1.33 ^d^	0.11 ± 0.01 ^d^
Light roasted Robusta	−6.03 ± 0.39 ^d^	−41.37 ± 1.39 ^d^	0.11 ± 0.01 ^d^
Dark roasted Robusta	−2.72 ± 0.45 ^f^	−40.95 ± 1.45 ^d^	0.12 ± 0.02 ^d^

Values are expressed as mean value ± SD; *n* = 6; ^a–g^ different letters in one column or no index correspond to significant differences (*p* < 0.05)

**Table 3:** Thermodynamic parameters of interactions between TPOIIα and fractions from coffee extracts before and after in vitro digestion measured by ITC.

Type of Coffee Fraction		∆H (kJ/mol)	∆G (kJ/mol)	∆S (J/mol×K)
Before in vitro digestion				
Green Arabica	Monochlorogenic acids	−1.28 ± 0.45 ^a^	−22.73 ± 1.45 ^a^	0.07 ± 0.00 ^a^
	Dichlorogenic acids	−1.25 ± 0.09 ^a^	−22.90 ± 1.02 ^a^	0.07 ± 0.00 ^a^
	Caffeine	−1.01 ± 0.15 ^a^	−22.73 ± 1.39 ^a^	0.07 ± 0.00 ^a^
Light roasted Arabica	Monochlorogenic acids	−1.26 ± 0.18 ^a^	−23.11 ± 1.33 ^a^	0.07 ± 0.00 ^a^
	Dichlorogenic acids	−1.24 ± 0.39 ^a^	−22.86 ± 1.45 ^a^	0.07 ± 0.00 ^a^
	Caffeine	−1.35 ± 0.45 ^a^	−29.39 ± 2.15 ^b^	0.09 ± 0.00 ^b^
Dark roasted Arabica	Monochlorogenic acids	−0.75 ± 0.01 ^b^	−23.24 ± 1.19 ^a^	0.07 ± 0.00 ^a^
	Dichlorogenic acids	−0.52 ± 0.05 ^b^	−19.51 ± 1.18 ^a^	0.06 ± 0.00 ^a^
	Caffeine	−1.01 ± 0.09 ^a^	−30.23 ± 2.21 ^c^	0.09 ± 0.00 ^b^
Green Robusta	Monochlorogenic acids	−2.40 ± 0.15 ^c^	−35.80 ± 2.25 ^d^	0.10 ± 0.00 ^b^
	Dichlorogenic acids	−1.44 ± 0.02 ^a^	−23.24 ± 1.19 ^a^	0.07 ± 0.00 ^a^
	Caffeine	−1.12 ± 0.10 ^a^	−22.52 ± 1.15 ^a^	0.07 ± 0.00 ^a^
Light roasted Robusta	Monochlorogenic acids	−1.35 ± 0.05 ^a^	−23.40 ± 2.26 ^a^	0.07 ± 0.00 ^a^
	Dichlorogenic acids	−1.37 ± 0.01 ^a^	−29.60 ± 3.21 ^b^	0.09 ± 0.00 ^b^
	Caffeine	−1.35 ± 0.01 ^a^	−29.43 ± 1.12 ^b^	0.09 ± 0.00 ^b^
Dark roasted Robusta	Monochlorogenic acids	−1.37 ± 0.01 ^a^	−29.56 ± 3.33 ^b^	0.09 ± 0.00 ^b^
	Dichlorogenic acids	−1.36 ± 0.02 ^a^	−29.52 ± 2.19 ^b^	0.09 ± 0.00 ^b^
	Caffeine	−1.35 ± 0.03 ^a^	−29.43 ± 2.22 ^b^	0.09 ± 0.00 ^b^
Gastric Phase				
Green Arabica	Monochlorogenic acids	−0.78 ± 0.02 ^b^	−33.14 ± 2.10 ^c^	0.10 ± 0.00 ^b^
	Dichlorogenic acids	−1.83 ± 0.01 ^a^	−29.54 ± 1.00 ^b^	0.09 ± 0.00 ^b^
	Caffeine	−1.60 ± 0.02 ^a^	−29.91 ± 2.20 ^b^	0.09 ± 0.00 ^b^
Light roasted Arabica	Monochlorogenic acids	−0.87 ± 0.02 ^b^	−32.84 ± 1.15 ^c^	0.10 ± 0.00 ^b^
	Dichlorogenic acids	−1.63 ± 0.01 ^a^	−29.83 ± 1.45 ^b^	0.09 ± 0.00 ^b^
	Caffeine	−1.52 ± 0.09 ^a^	−30.04 ± 1.39 ^c^	0.09 ± 0.00 ^b^
Dark roasted Arabica	Monochlorogenic acids	−0.46 ± 0.04 ^b^	−34.56 ± 2.28 ^c^	0.11 ± 0.00 ^b^
	Dichlorogenic acids	−0.48 ± 0.00 ^b^	−34.48 ± 3.25 ^c^	0.11 ± 0.00 ^b^
	Caffeine	−0.60 ± 0.01 ^b^	−33.85 ± 3.05 ^c^	0.11 ± 0.00 ^b^
Green Robusta	Monochlorogenic acids	−1.63 ± 0.09 ^a^	−29.83 ± 2.09 ^b^	0.09 ± 0.00 ^b^
	Dichlorogenic acids	−1.67 ± 0.12 ^a^	−29.79 ± 1.15 ^b^	0.09 ± 0.00 ^b^
	Caffeine	−1.78 ± 0.01 ^a^	−29.62 ± 1.11 ^b^	0.09 ± 0.00 ^b^
Light roasted Robusta	Monochlorogenic acids	−1.60 ± 0.10 ^a^	−29.91 ± 1.09 ^b^	0.09 ± 0.00 ^b^
	Dichlorogenic acids	−1.52 ± 0.09 ^a^	−29.54 ± 1.45 ^b^	0.09 ± 0.00 ^b^
	Caffeine	−1.67 ± 0.08 ^a^	−29.79 ± 1.33 ^b^	0.09 ± 0.00 ^b^
Dark roasted Robusta	Monochlorogenic acids	−0.52 ± 0.00 ^b^	−34.23 ± 1.19 ^c^	0.11 ± 0.00 ^b^
	Dichlorogenic acids	−0.34 ± 0.02 ^b^	−34.02 ± 1.45 ^c^	0.11 ± 0.00 ^b^
	Caffeine	−0.35 ± 0.01 ^b^	−32.97 ± 1.39 ^c^	0.10 ± 0.00 ^b^
Small Intestine Phase				
Green Arabica	Monochlorogenic acids	−16.91 ± 0.45 ^f^	−27.59 ± 1.95 ^b^	0.47 ± 0.01 ^c^
	Dichlorogenic acids	−17.21 ± 0.55 ^f^	−33.79 ± 1.81 ^c^	0.53 ± 0.02 ^c^
	Caffeine	−16.12 ± 0.39 ^f^	−26.92 ± 1.45 ^b^	0.87 ± 0.01 ^d^
Light roasted Arabica	Monochlorogenic acids	−14.00 ± 0.29 ^e^	−26.71 ± 1.55 ^b^	0.02 ± 0.00 ^a^
	Dichlorogenic acids	−15.85 ± 0.15 ^f^	−27.30 ± 1.39 ^b^	0.97 ± 0.03 ^d^
	Caffeine	−17.04 ± 0.10 ^f^	−33.70 ± 1.49 ^c^	0.97 ± 0.02 ^d^
Dark roasted Arabica	Monochlorogenic acids	−9.38 ± 0.02 ^d^	−27.42 ± 1.55 ^b^	0.26 ± 0.02 ^e^
	Dichlorogenic acids	−6.53 ± 0.09 ^d^	−23.70 ± 1.15 ^a^	0.42 ± 0.01 ^c^
	Caffeine	−12.73 ± 0.10 ^e^	−34.42 ± 1.25 ^c^	0.02 ± 0.00 ^a^
Green Robusta	Monochlorogenic acids	−30.14 ± 0.45 ^g^	−39.98 ± 1.35 ^d^	0.76 ± 0.01 ^f^
	Dichlorogenic acids	−18.07 ± 0.55 ^f^	−27.42 ± 1.49 ^b^	0.21 ± 0.02 ^e^
	Caffeine	−17.14 ± 0.39 ^f^	−33.75 ± 1.15 ^c^	0.59 ± 0.00 ^c^
Light roasted Robusta	Monochlorogenic acids	−16.98 ± 0.22 ^f^	−33.62 ± 1.25 ^c^	0.73 ± 0.0 ^f^
	Dichlorogenic acids	−15.51 ± 0.29 ^f^	−27.05 ± 1.35 ^b^	0.24 ± 0.01 ^e^
	Caffeine	−17.00 ± 0.15 ^f^	−33.62 ± 2.09 ^c^	0.66 ± 0.0 ^f^
Dark roasted Robusta	Monochlorogenic acids	−15.70 ± 0.39 ^f^	−27.09 ± 1.25 ^b^	0.77 ± 0.03 ^f^
	Dichlorogenic acids	−12.62 ± 0.05 ^e^	−26.92 ± 1.54 ^b^	0.16 ± 0.02 ^b^
	Caffeine	−16.98 ± 0.10 ^f^	−33.58 ± 2.19 ^c^	0.59 ± 0.01 ^c^
Large Intestine Phase after 4 h				
Green Arabica	Monochlorogenic acids	−2.21 ± 0.09 ^c^	−37.26 ± 1.15 ^d^	0.11 ± 0.00 ^b^
	Dichlorogenic acids	−1.52 ± 0.02 ^a^	−38.31 ± 1.59 ^d^	0.12 ± 0.00 ^b^
	Caffeine	−1.88 ± 0.01 ^a^	−37.72 ± 2.23 ^d^	0.12 ± 0.00 ^b^
Light roasted Arabica	Monochlorogenic acids	−2.16 ± 0.05 ^c^	−37.35 ± 1.09 ^d^	0.11 ± 0.00 ^b^
	Dichlorogenic acids	−1.65 ± 0.04 ^a^	−38.06 ± 1.21 ^d^	0.12 ± 0.00 ^b^
	Caffeine	−1.74 ± 0.01 ^a^	−37.93 ± 2.19 ^d^	0.12 ± 0.00 ^b^
Dark roasted Arabica	Monochlorogenic acids	−1.93 ± 0.02 ^a^	−37.64 ± 2.03 ^d^	0.12 ± 0.00 ^b^
	Dichlorogenic acids	−1.54 ± 0.03 ^a^	−38.27 ± 1.55 ^d^	0.12 ± 0.00 ^b^
	Caffeine	−1.77 ± 0.01 ^a^	−37.89 ± 1.65 ^d^	0.12 ± 0.00 ^b^
Green Robusta	Monochlorogenic acids	−3.81 ± 0.02 ^c^	−35.84 ± 1.36 ^d^	0.10 ± 0.00 ^b^
	Dichlorogenic acids	−8.16 ± 0.02 ^d^	−33.83 ± 2.29 ^c^	0.09 ± 0.00 ^b^
	Caffeine	−1.87 ± 0.01 ^c^	−37.72 ± 1.05 ^d^	0.12 ± 0.00 ^b^
Light roasted Robusta	Monochlorogenic acids	−2.46 ± 0.10 ^c^	−37.01 ± 1.95 ^d^	0.11 ± 0.00 ^b^
	Dichlorogenic acids	−3.86 ± 0.01 ^c^	−35.80 ± 1.15 ^c^	0.10 ± 0.00 ^b^
	Caffeine	−2.41 ± 0.15 ^c^	−37.05 ± 2.89 ^d^	0.11 ± 0.00 ^b^
Dark roasted Robusta	Monochlorogenic acids	−5.53 ± 0.45 ^e^	−34.88 ± 1.49 ^c^	0.10 ± 0.00 ^b^
	Dichlorogenic acids	−1.83 ± 0.15 ^a^	−37.81 ± 1.55 ^d^	0.12 ± 0.00 ^b^
	Caffeine	−1.91 ± 0.01 ^a^	−37.68 ± 1.75 ^d^	0.11 ± 0.00 ^b^
Large Intestine Phase after 10 h				
Green Arabica	Monochlorogenic acids	−0.75 ± 0.00 ^b^	−32.95 ± 1.39 ^c^	0.09 ± 0.00 ^b^
	Dichlorogenic acids	−1.13 ± 0.09 ^a^	−28.54 ± 1.05 ^b^	0.09 ± 0.00 ^b^
	Caffeine	−1.25 ± 0.05 ^a^	−28.91 ± 1.10 ^b^	0.09 ± 0.00 ^b^
Light roasted Arabica	Monochlorogenic acids	−0.55 ± 0.02 ^b^	−31.84 ± 1.02 ^c^	0.10 ± 0.00 ^b^
	Dichlorogenic acids	−1.12 ± 0.02 ^a^	−28.83 ± 1.01 ^b^	0.09 ± 0.00 ^b^
	Caffeine	−1.19 ± 0.02 ^a^	−29.04 ± 1.43 ^c^	0.09 ± 0.00 ^b^
Dark roasted Arabica	Monochlorogenic acids	−0.05 ± 0.01 ^b^	−33.56 ± 2.35 ^c^	0.11 ± 0.00 ^b^
	Dichlorogenic acids	−0.01 ± 0.01 ^b^	−33.48 ± 2.12 ^c^	0.11 ± 0.00 ^b^
	Caffeine	−0.08 ± 0.00 ^b^	−32.85 ± 2.15 ^c^	0.11 ± 0.00 ^b^
Green Robusta	Monochlorogenic acids	−1.73 ± 0.03 ^a^	−28.83 ± 1.09 ^b^	0.09 ± 0.00 ^b^
	Dichlorogenic acids	−1.55 ± 0.09 ^a^	−28.79 ± 1.21 ^b^	0.09 ± 0.00 ^b^
	Caffeine	−1.38 ± 0.05 ^a^	−28.62 ± 1.35 ^b^	0.09 ± 0.00 ^b^
Light roasted Robusta	Monochlorogenic acids	−1.18 ± 0.03 ^a^	−28.91 ± 1.15 ^b^	0.09 ± 0.00 ^b^
	Dichlorogenic acids	−1.52 ± 0.05 ^a^	−28.54 ± 1.12 ^b^	0.09 ± 0.00 ^b^
	Caffeine	−1.14 ± 0.03 ^a^	−28.79 ± 1.24 ^b^	0.09 ± 0.00 ^b^
Dark roasted Robusta	Monochlorogenic acids	−0.45 ± 0.01 ^b^	−33.23 ± 1.23 ^c^	0.11 ± 0.00 ^b^
	Dichlorogenic acids	−0.26 ± 0.00 ^b^	−33.02 ± 1.39 ^c^	0.11 ± 0.00 ^b^
	Caffeine	−0.11 ± 0.00 ^b^	−31.97 ± 1.05 ^c^	0.10 ± 0.00 ^b^
Large Intestine Phase + probiotic bacteria after 4 h				
Green Arabica	Monochlorogenic acids	−11.28 ± 0.49 ^e^	−31.78 ± 1.05 ^c^	0.18 ± 0.00 ^b^
	Dichlorogenic acids	−10.75 ± 0.55 ^e^	−31.11 ± 1.17 ^c^	0.73 ± 0.03 ^f^
	Caffeine	−9.34 ± 0.15 ^d^	−30.90 ± 2.23 ^c^	0.61 ± 0.03 ^f^
Light roasted Arabica	Monochlorogenic acids	−10.47 ± 0.15 ^e^	−31.28 ± 1.26 ^c^	0.18 ± 0.02 ^b^
	Dichlorogenic acids	−8.42 ± 0.45 ^d^	−31.11 ± 2.24 ^c^	0.26 ± 0.02 ^e^
	Caffeine	−11.32 ± 0.49 ^e^	−37.76 ± 2.35 ^d^	0.38 ± 0.01 ^e^
Dark roasted Arabica	Monochlorogenic acids	−6.25 ± 0.32 ^d^	−31.61 ± 2.58 ^c^	0.87 ± 0.01 ^d^
	Dichlorogenic acids	−4.35 ± 0.33 ^c^	−27.88 ± 2.65 ^b^	0.96 ± 0.02 ^d^
	Caffeine	−10.56 ± 0.39 ^d^	−31.48 ± 1.22 ^c^	0.54 ± 0.01 ^c^
Green Robusta	Monochlorogenic acids	−20.10 ± 0.01	−44.17 ± 2.55 ^e^	0.72 ± 0.012 ^f^
	Dichlorogenic acids	−12.04 ± 0.05 ^e^	−31.61 ± 1.94 ^c^	0.17 ± 0.00 ^b^
	Caffeine	−11.47 ± 0.10 ^e^	−37.97 ± 2.87 ^d^	0.56 ± 0.02 ^c^
Light roasted Robusta	Monochlorogenic acids	−11.33 ± 0.11 ^e^	−37.81 ± 1.45 ^d^	0.65 ± 0.02 ^f^
	Dichlorogenic acids	−8.49 ± 0.02 ^d^	−38,60. ± 1.98 ^d^	0.23 ± 0.01 ^e^
	Caffeine	−11.43 ± 0.05 ^e^	−37.93 ± 2.22 ^d^	0.56 ± 0.01 ^c^
Dark roasted Robusta	Monochlorogenic acids	−11.36 ± 0.01 ^e^	−37.89 ± 1.39 ^d^	0.65 ± 0.012 ^f^
	Dichlorogenic acids	−10.56 ± 0.15 ^e^	−31.48 ± 1.55 ^c^	0.54 ± 0.01 ^c^
	Caffeine	−11.32 ± 0.10 ^d^	−37.81 ± 1.25 ^d^	0.52 ± 0.01 ^c^
Large Intestine Phase + probiotic bacteria after 10 h				
Green Arabica	Monochlorogenic acids	−7.62 ± 0.39 ^d^	−34.04 ± 1.05 ^c^	0.09 ± 0.00 ^b^
	Dichlorogenic acids	−1.81 ± 0.33 ^a^	−37.85 ± 1.22 ^d^	0.12 ± 0.00 ^b^
	Caffeine	−8.16 ± 0.45 ^d^	−33.83 ± 2.29 ^c^	0.08 ± 0.00 ^a^
Light roasted Arabica	Monochlorogenic acids	−1.69 ± 0.05 ^a^	−38.02 ± 2.39 ^d^	0.12 ± 0.00 ^b^
	Dichlorogenic acids	−1.56 ± 0.02 ^a^	−38.23 ± 1.18 ^d^	0.12 ± 0.00 ^b^
	Caffeine	−2.62 ± 0.45 ^c^	−36.84 ± 1.45 ^d^	0.11 ± 0.00 ^b^
Dark roasted Arabica	Monochlorogenic acids	−1.39 ± 0.03 ^a^	−38.56 ± 1.35 ^d^	0.12 ± 0.00 ^b^
	Dichlorogenic acids	−1.44 ± 0.02 ^a^	−38.48 ± 2.45 ^d^	0.12 ± 0.00 ^b^
	Caffeine	−8.37 ± 0.11 ^d^	−33.79 ± 2.49 ^c^	0.08 ± 0.00 ^a^
Green Robusta	Monochlorogenic acids	−9.13 ± 0.22 ^d^	−37.14 ± 1.39 ^d^	0.11 ± 0.00 ^b^
	Dichlorogenic acids	−9.17 ± 0.09 ^d^	−33.54 ± 1.61 ^c^	0.08 ± 0.00 ^a^
	Caffeine	−7.00 ± 0.25 ^d^	−33.91 ± 1.75 ^c^	0.08 ± 0.00 ^a^
Light roasted Robusta	Monochlorogenic acids	−8.16 ± 0.12 ^d^	−33.83 ± 2.82 ^c^	0.08 ± 0.00 ^a^
	Dichlorogenic acids	−8.33 ± 0.09 ^d^	−33.79 ± 2.55 ^c^	0.08 ± 0.00 ^a^
	Caffeine	−8.92 ± 0.45 ^d^	−33.62 ± 1.55 ^c^	0.08 ± 0.00 ^a^
Dark roasted Robusta	Monochlorogenic acids	−8.00 ± 0.57 ^d^	−33.91 ± 2.59 ^c^	0.08 ± 0.00 ^a^
	Dichlorogenic acids	−2.35 ± 0.49 ^c^	−33.54 ± 1.49 ^c^	0.08 ± 0.00 ^a^
	Caffeine	−2.49 ± 0.25 ^c^	−35.97 ± 1.55 ^d^	0.11 ± 0.00 ^b^

Values are expressed as mean value ± SD; *n* = 6; ^a–g^ different letters in one column or no index correspond to significant differences (*p* < 0.05)

**Table 4:** Molecular docking results from AutoDock for mixtures of main ligands from a fraction and TOPIIα. Docking performed in different sequences of the ligands.

Sequence	∆G (kJ/mol)	Interaction		
		Hydrogen	Hydrophobic	Salt Bridges
3CQA; 4CQA; 5CQA	−47.61	Thr49 (2.25 Å, 1.93 Å), Arg162 (2.71 Å, 1.94 Å), Asn163 (2.04 Å), Pro371 (2.14 Å, 2.09 Å), Phe373 (1.94 Å, 1.98 Å), Gln376 (1.99 Å)	Arg162 (3.47 Å), Tyr165 (3.66 Å), Thr372 (3.81 Å), Gln376 (3.93)	Arg162 (3.66 Å)
3CQA; 5CQA; 4CQA	−47.61	Asp48 (2.04 Å), Pro79 (2.04 Å), Tyr82 (1.84 Å), Lys (2.19 Å), Ala318 (2.16 Å, 2.76 Å), Ser320 (2.86 Å), Thr377 (2.74 Å), Glu379 (1.99 Å)	Pro79 (3.58 Å), Ala318 (3.59 Å), Thr377 (3.26 Å),	Lys83 (3.92 Å), Arg241 (3.72 Å)
4CQA; 3CQA; 5CQA	−46.82	Pro79 (2.06 Å), Lys321 (3.51 Å), Asp374 (2.29 Å), Thr377 (1.87 Å), Glu379 (2.28 Å), Asn380 (2.38 Å)	Pro79 (3.67 Å), Lys83 (3.63 Å), Asp374 (3.35 Å), Thr377 (3.18 Å)	Lys321 (2.56 Å)
4CQA; 5CQA; 3CQA	−46.82	Pro79 (1.93 Å), Asp374 (2.17 Å), Thr377 (1.83 Å), Glu379 (2.18 Å)	Pro79 (3.60 Å), Lys83 (3.60 Å), Asp (3.29 Å), Thr377 (3.31 Å)	Lys321 (2.63 Å, 4.17 Å)
5CQA; 3CQA; 4CQA	−44.52	Pro79 (1.89 Å), Tyr82 (2.98 Å; 2.39 Å, 3.39 Å), Ala318 (1.93 Å), Lys321 (2.05 Å), Thr377 (3.08)	Pro79 (3.50 Å), Asp86 (3.38 Å), Thr377 (3.57 Å)	Lys83 (3.71 Å, 4.75 Å), Arg242 (4.10 Å)
5CQA; 4CQA; 3CQA	−44.52	Ile51 (1.74 Å), Tyr82 (3.12 Å), Lys83 (3.43 Å), Asp86 (2.90 Å), Arg241 (3.44 Å), Ala318 (3.36 Å), Lys321 (2.91 Å), Ser375 (2.03 Å), Thr377 (2.04 Å, 2.14 Å), Glu379 (3.00 Å, 2.10 Å)	Tyr82 (3.40 Å, 3.68 Å), Thr377 (3.42 Å),	-
3,4-DCQA; 3,5,-DCQA; 4,5-DCQA	−54.64	Asp94 (1.86 Å), Gln97 (3.08 Å), Asn150 (2.48 Å), Glu155 (2.44 Å), Lys156 (2.34 Å), Lys157 (2.07 Å), Val158 (2.06 Å)	Asp94 (3.34 Å), Arg98 (3.57 Å) Lys157 (3.42 Å), Thr159 (3.26 Å)	Arg98 (5.26 Å), Lys157 (4.11 Å, 4.17 Å)
3,4-DCQA; 4,5,-DCQA; 3,5-DCQA	−54.64	Gln97 (2.79 Å, 2.93 Å), Arg98 (2.69 Å, 2.28 Å), Ser149 (2.45 Å), Tyr151 (3.30 Å), Lys157 (1.95 Å), Val158 (1.84 Å)	Gln97 (3.57 Å), Arg98 (3.45 Å), Lys157 (3.83 Å, 3.62 Å), Thr159 (3.33 Å)	Arg98 (5.14 Å) Lys157 (3.67 Å)
3,5-DCQA; 3,4,-DCQA; 4,5-DCQA	−48.70	Glu133 (2.85 Å, 1.95 Å), Asp162 (1.96 Å, 1.95 Å)	Ile125 (3.32 Å), Val137 (3.19 Å), Leu140 (3.26 Å), Thr147 (3.21 Å)	Agr98 (3.02 Å), Lys157 (5.22 Å)
3,5-DCQA; 4,5,-DCQA; 3,4-DCQA	−48.70	Ser148 (1.95 Å), Tyr151 (2.13 Å, 3.16 Å), Lys156 (2.79 Å), Val158 (2.16 Å)	Gln97 (3.99 Å), Asp152 (3.26 Å), Lys157 (3.68 Å)	Agr98 (3.20 Å, 5.34 Å), Lys157 (4.33 Å, 4.74 Å)
4,5-DCQA; 3,4,-DCQA; 3,5-DCQA	−56.53	Gln97 (3.14 Å, 3.01 Å), Arg98 (2.72 Å, 2.26 Å), His130 (1.83 Å), Ser149 (2.42 Å), Lys157 (2.27 Å), Val158 (1.92 Å)	Gln97 (3.67 Å), Arg98 (3.52 Å), Leu140 (3.66 Å), Ile141 (3.13 Å), Thr147 (3.99 Å), Lys157 (3.91 Å, 3.62 Å), Thr159 (3.39 Å)	Arg98 (5.35 Å), Lys157 (3.75 Å)
4,5-DCQA; 3,5,-DCQA; 3,4-DCQA	−56.53	Leu146 (1.95 Å), Ser148 (3.34 Å, 3.03 Å), Tyr151 (2.45 Å), Asp152 (2.87 Å, 2.06 Å), Glu155 (1.89 Å, 2.18 Å)	Val137 (3.29 Å), Ile141 (3.63 Å), Tyr151 (3.29 Å)	His130 (5.06 Å)

## Data Availability

Data available on request.
